# Influence of medium modifications (optimization) on high nematicidal activity of the fermentation broth of *Clostridium beijerinckii*

**DOI:** 10.3389/fbioe.2023.1283112

**Published:** 2024-01-03

**Authors:** Shuang Liu, Kejun Zhang, Yun Yu, Xinglong Lian, Lanyuwen Jiang, Fanqi Meng, Yuanyuan Wang, Xiaofeng Zhu, Yuxi Duan

**Affiliations:** ^1^ College of Bioscience and Biotechnology, Shenyang Agricultural University, Shenyang, China; ^2^ Tianjin Vocational College of Bioengineering, Tianjin, China; ^3^ Nematology Institute of Northern China, Shenyang Agricultural University, Shenyang, China

**Keywords:** *Meloidogyne incognita*, *Clostridium beijerincki*i, optimization, response surface methodology, nematicidal, fermentation, mortality

## Abstract

**Introduction:** The nematode species *Meloidogyne incognita* has been responsible for significant financial losses within the agricultural sector. Nematophagous bacteria, characterised by their extensive distribution and broad spectrum of hosts, exhibit remarkable efficacy as natural antagonists against nematodes. Sneb518 (*Clostridium beijerinckii*) fermentation broth displayed substantial biocontrol activity against *M. incognita* in previous research. Optimizing fermentation conditions is a fundamental technique for dramatically enhancing end product performance. There has been no such study conducted yet on enhancing the nematicidal activities of Sneb518 (*Clostridium beijerinckii*) fermentation using response surface methodology (RSM).

**Methods:** The influence of strain Sneb518 fermentation media and conditions on nematicidal activity was examined using the three-factor technique and a Plackett-Burman design, and the interaction between various fermentation factors was examined using a Box-Behnken design. The present study employed response surface methodology (RSM) to examine and enhance the nematicidal activity of Sneb518 culture filtrates by identifying and optimising the influential components.

**Results:** Glucose, peanut cake flour, and potassium chloride as carbon, nitrogen, and inorganic salts displayed considerably increased nematicidal potential in the present study. Furthermore, the corrected mortality of J2 ranged from 52.24% to 91.15% when utilizing the Box-Behnken design. These findings clearly support the application of RSM for medium optimization. Moreover, the outcomes of the validation experiment corresponded to the model predictions.

**Discussion:** This research has enhanced the biocontrol ability of *C. beijerinckii* to control *M. incognita* and this research has led to the advancement of new biocontrol agents.

## 1 Introduction

Plant parasitic nematodes (PPNs) are one of the most prevalent pathogenic organisms responsible for disease in plants (Sikandar et al., 2023). Among them, the root-knot nematode is among the greatest destructive PPNs (Sikandar et al., 2019). However, there are few types of targeted effective agents, and several effective chemical nematicides are restricted or banned due to their high toxicity and high residue levels, so the search for high-efficiency, low-toxicity compounds from microbial sources destroying PPNs has become an important research focus (Sikandar et al., 2020). Bacteria are favored for their abundant resources and complex and novel active ingredients in metabolites (Wang et al., 2020). Research on bacterial metabolites reveals that the level of antibiotics produced by fermentation depends on not only the bacterial strain but also the suitable environmental conditions given for expressing its production capacity, and these two factors are complementary. Therefore, fermentation optimization plays a decisive role in enhancing the production of specific metabolites.

To achieve the optimal circumstances where each variable coordinates with other factors to provide the best expected outcomes, the Plackett–Burman experimental design allows for the randomization of many variables (Plackett and Burman, 1946). The outcome of the surface approach may create a constantly changing surface model while concurrently determining the number of elements that impact yield and their levels (Latha et al., 2017). Interactions are optimized and evaluated, so optimal conditions for multifactorial systems can be determined quickly and efficiently (Yin and Wang, 2019). There are a wide range of applications for optimizing the fermentation media to increase their nematicidal potentials (Zhang et al., 2020; Abd-Elgawad, 2021).

In this experiment, a novel nematicide bacterial strain Sneb518 was selected. As a result, the purpose of this research is to screen for and optimize the ideal fermentation medium using the surface response approach in order to achieve the best parameters of the fermentation process of strain Sneb518 so as to improve the yield of nematicide active substances and lay the foundation for the separation and purification of active substances produced by the strains.

## 2 Materials and methods

### 2.1 Preparation of J2s of *Meloidogyne incognita*

*Meloidogyne incognita* was used in all experiments and cultivated on the susceptible tomato cultivar L402 within a controlled greenhouse environment at Shenyang Agricultural University, China. To minimize potential microbial contamination, microscopically selected egg masses of *M. incognita* were surface-sterilized with 0.5% sodium hypochlorite solution for 3 min and then washed three times with sterile water before incubation at 28°C to facilitate the hatching of J2s (Sikandar et al., 2021).

### 2.2 Preparation of the strain and seed fermentation broth

*Clostridium beijerinckii* Sneb518 was cultured on beef extract peptone agar medium (NA) plates, then floated in sterile distilled water, and diluted to 1.0 × 10^8^ cfu/mL using a hemocytometer under a microscope. The diluted bacteria were transferred to 500 mL of sterilized lysogeny broth (LB). The cultures were incubated at 28°C and 180 rpm for 48 h and subsequently used as a seed fermentation broth for future experiments (Lian et al., 2022).

### 2.3 Medium modifications of Sneb518 in anaerobic fermentation

The optimal nutrient sources, including carbon supply, nitrogen supply, and inorganic salts, were determined by a one-factor-at-a-time approach in anaerobic fermentation.

The effects of nutrient substances are stated in reference to a nematicidal activity based on LB. The effects of nutrient substances on Sneb518 fermentation were examined in 250-mL Erlenmeyer flasks having an effective quantity of 100 mL LB. Eleven carbon sources (maltose, lactose, mannitol, sucrose, glucose, galactose, sorbitose, corn starch, soluble starch, fructose, and ribose), 10 nitrogen sources (ammonium chloride, ammonium dihydrogen phosphate, ammonium nitrate, ammonium sulfate, protein, beef paste, yeast paste, soybean powder, urea, and peanut cake powder), and 12 inorganic salts (calcium chloride, potassium dihydrogen phosphate, sodium dihydrogen phosphate, sodium chloride, potassium chloride, disodium hydrogen phosphate, dipotassium hydrogen phosphate, calcium carbonate, magnesium chloride, magnesium sulfate, zinc chloride, and zinc sulfate) were selected based on the nematicidal activity under different culture conditions. Based on the LB medium, a single additional concentration of 1% nitrogen, 1% carbon, and 0.5% inorganic salt was added, respectively. The nitrogen and carbon sources and inorganic salts were added to the medium at the corresponding ratios, respectively, and then inoculated with 1 mL of seed fermentation broth and culture under anaerobic conditions. The anaerobic chamber with a slightly modified atmosphere of 15% CO_2_, 82% N_2_, and 3% H_2_ was employed to finish all inoculations, handling in anaerobic fermentation, and incubation processes at 28°C for 24 h. Before fermentation, the LB medium was stored in the anaerobic chamber overnight with loose caps on bottles and test tubes to remove any remaining oxygen. According to the nematicidal activity, the more suitable nutrient substance was chosen.

### 2.4 Screening of the medium components of Sneb518 by the Plackett–Burman Technique

The nematicidal activity of the Sneb518 broth was investigated using a Plackett–Burman experimental design to screen the important medium components using a one-factor-at-a-time method. Six different factors were taken into account throughout the screening experiments: galactose, glucose, soybean powder, ammonium dihydrogen phosphate, sodium dihydrogen phosphate, and calcium chloride. Table 1 shows the range of values for each variable, with high values represented by (+1) and low values by (−1). Twelve tests were conducted using the Plackett–Burman method. Table 1 displays the coding levels and real-world values of the variables and the design matrix. Design-Expert 8.1.1 was used for data processing and analysis of the tests. All experiments were run in triplicate, and the results were expressed as the mean standard deviation. The Box–Behnken design was used to further optimize the factors with more than 95% confidence levels, which were believed to substantially influence the nematicidal activity of the Sneb518 broth (Table 1).

**TABLE 1:** Factors and levels of Sneb518 medium composition using the Plackett–Burman design.

Symbol code	Variable	Levels	
		Low/−1	High/+1
A	Glucose (g/L)	0.5	2.5
B	Peanut cake flour (g/L)	0.5	2.5
C	Sodium hydrogen phosphate (g/L)	0.1	1
D	Ribose (g/L)	0.5	2.5
E	Beef extract (g/L)	0.5	2.5
F	Potassium chloride (g/L)	0.1	1

### 2.5 Optimization of screened components using the Box–Behnken design

We used a Box–Behnken factorial design with three components and three levels to maximize the nematicidal activity of the Sneb518 broth (Shah et al., 2022). The model featured three duplicated center points and a set of points that characterized the area of interest and was used to fit a second-order response surface at the midpoints of each edge of the multidimensional cube. Using this experimental design, the influence of the screening medium ingredients (glucose, peanut cake flour, and sodium dihydrogen phosphate) on the expression level of nematicidal activity was assessed. The three screening factors were labeled A, B, and C, respectively, and adjusted mortality was labeled R, which is a response. Table 2 shows the levels of each variable. To assess pure effort, all studies were done with three replications at the center positions. Validation trials were used to confirm the optimal settings. The responses were tracked and compared to what the model anticipated (Yasin et al., 2014; Kumar et al., 2015). To visualize the relationship between the response and experimental levels of each element and to determine the best condition, the fitted polynomial equation was represented as response plots using Minitab (version 16) software.

**TABLE 2:** Factors and levels of Sneb518 medium composition using the Box–Behnken experimental design.

Number	Factor	Coded values		
		Low ^−1^	Middle^0^	High ^+1^
A	Glucose (g/L)	0.5	1	1.5
B	Peanut cake flour (g/L)	0.5	1	1.5
C	Sodium dihydrogen phosphate (g/L)	0.1	0.3	0.5

### 2.6 Nematicidal activity assay using the broth with pre- and post-medium modifications

Screening of nematicidal activity of the Sneb518 (*C. beijerinckii*) fermentation broth was operated according to methods proposed by Lian et al. (2022) using the broth with pre- and post-medium modifications. Approximately 100 newly hatched *M. incognita* J2s were placed in 5-mL glass Petri dishes along with 2 mL of fermentation broth to determine their impact on J2 mortality. The control treatment consisted of using sterile water. The tubes were incubated in the dark at 26°C. The number of hatching J2s was counted using a stereomicroscope. The experiment was repeated three times, with three replicates in each experiment. The experiment was conducted in triplicate, with three duplicates. The below equation was used to compute the nematicidal efficacy:

$$\text{Corrected mortality }\left(\%\right)=\frac{\text{Number}\;\text{of}\;\text{dead}\;J2s\;\text{in}\;\text{treatment}\;–\;\text{Number}\;\text{of}\;\text{dead}\;J2s\;\text{in}\;\text{CK}}{1-\;\text{No.of dead J2s in CK}}.$$ (1)

### 2.7 Statistical analysis

SPSS software (version 20.0) was used to conduct all statistical analyses. To evaluate the significant difference between the treatments, Duncan’s multiple range test (*p* < 0.05) was utilized.

## 3 Results

### 3.1 Medium modifications of Sneb518 in anaerobic fermentation

**FIGURE 1:**
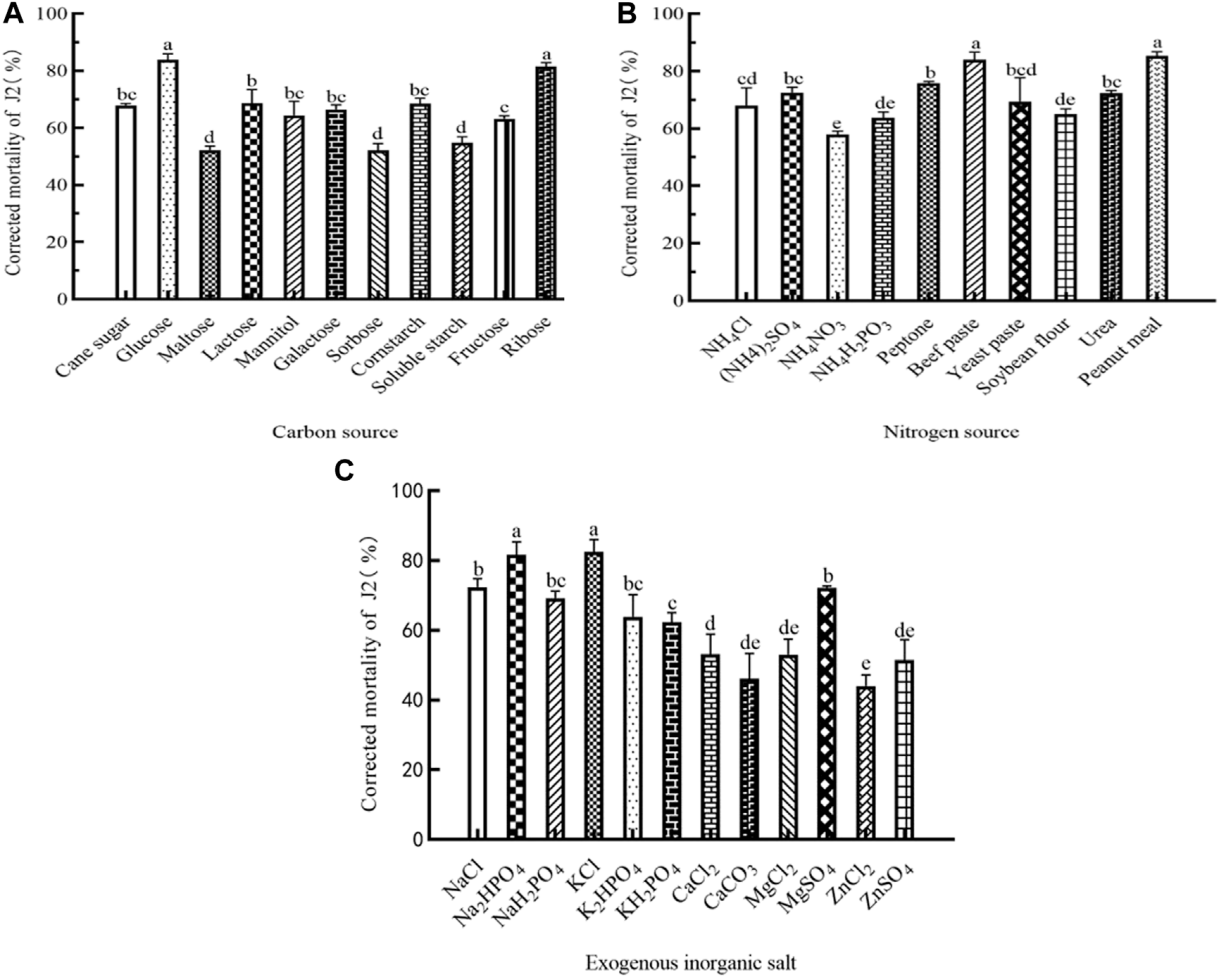
Medium components of Sneb518 strain fermentation on the corrected mortality of root-knot nematode J2. **(A)** Carbon source; **(B)** nitrogen source; and **(C)** exogenous inorganic salt. The different letters indicate that values are significantly different according to Duncan’s multiple range test at *p* < 0.05.

To evaluate the higher nematicidal activity, it is crucial to investigate the importance of each medium component and their interaction. Therefore, experiments were conducted to identify the significant medium components and optimize their concentrations. The outcomes of the one-factor-at-a-time method revealed that Sneb518 had different nutritional species requirements under anaerobic conditions. The fermentation process utilizes various nutrients and exhibits diverse nematicidal activity. When tested under anaerobic conditions, glucose was found to have the highest impact on the nematicidal activity of the fermentation broth (83.77%), followed by ribose (81.46%). The mortality rate was significantly improved by 85.25% when peanut cake powder was used as a nitrogen source. Similarly, when beef extract was employed as a nitrogen source, a large benefit was observed, with an approximate increase of 84%. Furthermore, the addition of potassium chloride and disodium hydrogen phosphate to the fermentation broth led to substantial increases in its nematicidal activity. Specifically, the nematicidal activity increased by 82.54% and 81.53%, respectively, in the current research (Figure 1).

### 3.2 Deeper insight into the medium components of Sneb518 by The Plackett–Burman Technique

The Plackett–Burman investigations were conducted following the design matrix provided in Table 3. The corrected mortality varied from 47.15% to 85.37%. This considerable variance in nematicidal activity underlined the necessity of optimizing medium ingredients. The analysis of variance (ANOVA) was used for selecting the relevant components, and the importance of the variables was determined using Duncan’s multiple range test (Table 4). Multiple regression analysis was performed to validate the model, and the first-order polynomial was reported as expressed in Eq. 2:

$$Y_{1}=7.11A-8.15B+5.73C-0.89D+1.09E-1.38F+62.41,$$ (2)

where Y_1_ = corrected mortality (%), A = glucose (g/L), B = peanut cake flour (g/L), C = sodium dihydrogen phosphate (g/L), D = ribose (g/L), E = beef extract (g/L), and F = potassium chloride (g/L).

**TABLE 3:** Plackett–Burman experimental design and results of Sneb518 medium components.

Number	A	B	C	D	E	F	Corrected mortality (%)
1	1	1	−1	1	1	1	53.54 ± 5.51 ef
2	−1	1	1	−1	1	1	47.15 ± 3.48 g
3	1	−1	1	1	−1	1	81.35 ± 3.11 fg
4	−1	1	−1	1	1	−1	48.68 ± 1.71 d
5	−1	−1	1	−1	1	1	76.11 ± 0.74 b
6	−1	−1	−1	1	−1	1	50.75 ± 3.21 fg
7	1	−1	−1	−1	1	−1	70.18 ± 2.03 c
8	1	1	−1	−1	−1	1	57.29 ± 1.63 de
9	1	1	1	−1	−1	−1	69.44 ± 2.73 c
10	−1	1	1	1	−1	−1	49.64 ± 2.61 fg
11	1	−1	1	1	1	−1	85.37 ± 1.86 a
12	−1	−1	−1	−1	−1	−1	59.65 ± 1.82 d

**TABLE 4:** ANOVA analysis of Plackett–Burman experiment data.

Source	SS	DF	MS	F	*p*
Model	1846.27	6	307.71	8.81	0.0152*
Glucose	607.08	1	607.08	17.38	0.0087**
Peanut cake flour	797.93	1	797.93	22.84	0.005**
Sodium hydrogen phosphate	394.59	1	394.59	11.3	0.0201*
Ribose	9.46	1	9.46	0.27	0.625
Beef extract	1.43	1	1.43	0.41	0.5508
Potassium chloride	22.94	1	22.94	0.66	0.4545
Residual (error)	174.65	5	3.49	—	—
Total	2020.92	11	—	—	—

Note:* indicates significant difference at *p* < 0.05 using Duncan’s multiple range test.

All experiments were run in triplicate, and the results were expressed as the mean standard deviation. Significant influences on the adjusted mortality rate were attributed to the factors with significance levels more than 95% (*p* < 0.05). In the present study, glucose, peanut cake flour, and sodium hydrogen phosphate displayed significance (*p* < 0.05) and were regarded as important factors for further optimization research employing the Plackett–Burman design (Table 4).

### 3.3 Optimization of screened components using the Box–Behnken design

To assess and analyze the impact of screening factors on the corrected mortality of J2, we employed a three-level Box–Behnken design. The design matrix and its corresponding findings are provided in Table 5. The corrected mortality of J2 ranged from 52.24% to 91.15%.

**TABLE 5:** Box–Behnken experimental design and results of Sneb518 medium composition.

Number	A	B	C	Corrected mortality (%)
1	0	0	0	91.15 ± 0.10 a
2	1	0	−1	62.69 ± 6.03 e
3	0	0	0	90.27 ± 2.60 a
4	0	−1	1	71.75 ± 6.11 cd
5	−1	−1	0	74.63 ± 2.87 bc
6	0	0	0	92.51 ± 8.79 a
7	1	1	0	80.55 ± 2.80 b
8	−1	0	−1	75.97 ± 3.62 bc
9	1	−1	0	79.73 ± 3.62 b
10	0	0	0	90.08 ± 1.63 a
11	−1	0	1	52.24 ± 7.96 f
12	0	1	−1	66.01 ± 4.54 de
13	0	−1	−1	74.13 ± 1.29 bc
14	−1	1	0	80.02 ± 5.07 b
15	0	0	0	88.43 ± 2.49 a
16	0	1	1	71.74 ± 3.11 cd
17	1	0	1	76.54 ± 2.42 bc

Data are given as the mean standard deviation from two replicates of each experiment. It was determined that the factors with significance levels more than 95% (*p* < 0.05) exhibited a substantial effect on the corrected mortality rate. In the present study, AC, A^2^, B^2^, and C^2^ displayed significance (*p* < 0.05) and were identified as important variables for subsequent optimization investigations using the Box–Behnken design (Table 6).

**TABLE 6:** Simulation coefficient evaluation and significance test of the Sneb518 medium composition regression equation.

Source	SS	DF	MS	F	*p*
Model	2681.58	9	297.95	8.09	0.0058**
A	54.71	1	54.71	1.48	0.2625
B	11.09	1	11.09	0.3	0.6003
C	58.21	1	58.21	1.58	0.2491
AB	79.66	1	79.66	2.16	0.1849
AC	298.08	1	298.08	8.09	0.0249*
BC	0.95	1	0.95	0.026	0.8769
A^2^	622.57	1	622.57	16.9	0.0045**
B^2^	1061.75	1	1061.75	28.82	0.001**
C^2^	283.79	1	283.79	7.7	0.0275*
Residual (error)	257.88	7	36.84	—	—
Lack of fit	204.59	3	68.2	5.12	0.0743
Pure error	53.29	4	13.32	—	—
Total	2939.46	16	—	—	—

Note:* indicates significant difference at *p* < 0.05 using Duncan’s test.

Multiple regression analysis was used to test the model suitability, and Eq. 3 was fitted using a second-order polynomial model as a result:

$$Y_{2}=90.55+2.08A-0.24B-0.82C-1.14\text{AB}+9.40\text{AC}+2.03\text{BC}-7.93A^{2}-3.88B^{2}-15.76C^{2},$$ (3)

where Y_2_ = corrected mortality (%), A = glucose (g/L), B = peanut cake flour (g/L), and C = sodium dihydrogen phosphate (g/L).

**FIGURE 2:**
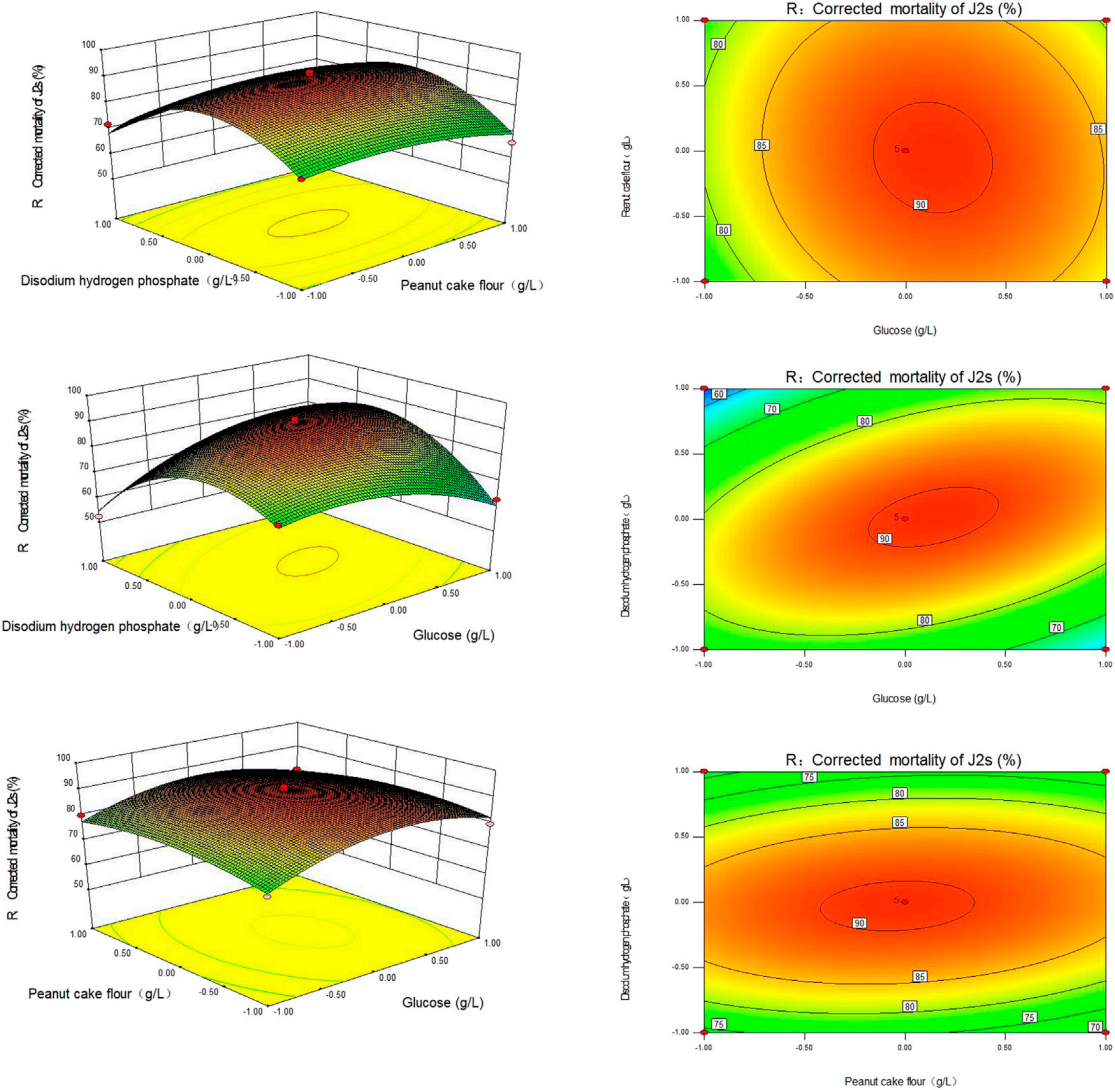
Response surface and contour plot of the Sneb518 fermentation broth medium. **(A)** Glucose and peanut cake powder; **(B)** glucose and disodium hydrogen phosphate; and **(C)** peanut cake powder and disodium hydrogen phosphate.

The corrected mortality was calculated using the 3D response plots created for various tested variable values. As illustrated in Figure 2, the plot was constructed such that the response was displayed on the *z*-axis against either two independent variables while keeping the remaining variables at their optimal values.

## 4 Discussion

Optimization of fermentation conditions is a critical strategy for significantly increasing the end product (Singh et al., 2017). Numerous biocontrol bacteria and fungi can produce secondary metabolites that can effectively inhibit plant pathogenic nematodes. However, the production capacity of these strains is often low and unstable in their natural state. This poses a significant challenge for large-scale industrial production. Therefore, enhancing the necessary product level in the fermentation broth of production strains has become a crucial concern. The interaction between various factors was investigated, and the optimal conditions were obtained.

With more than 100 species, *Clostridium* is the second largest genus after *Streptomyces* (Dong et al., 2010). *Clostridium* species can ferment a broad range of substrates, including glycerol, disaccharides, hexoses, syngas, starch, pentoses, and cellulose, into fuels and chemicals that are sustainable and useful to industry (Liberato et al., 2019). Studies demonstrated that some species of *Clostridium* can stimulate plant growth (Polyanskaya et al., 2002; Lian et al., 2022). *Clostridium* bacteria produce toxins that are lethal to nematodes (Aktories et al., 2012). Strains of *C. beijerinckii* Sneb518 may have a biocontrol impact since they stimulate plant growth and secrete compounds that kill nematode J2s and prevent egg hatching (Lian et al., 2022).

Under various culture conditions, microbes may produce different inhibitory compounds (Couto et al., 2017). The results of this experiment showed that fermentation products of *C. beijerinckii* had a greater effectiveness in reducing nematodes when the carbon source in the medium was glucose. This could be attributed to the fact that monosaccharides are easier for the organism to absorb and utilize, leading to the production of inhibitory substances compared to disaccharides and polysaccharides. Our finding is in line with those obtained by Pan et al. (2023) who optimized glucose as a carbon source for *Bacillus pumilus* LYMC-3 fermentation to improve the synthesis of inhibitory substances. Similarly, in *Streptomyces tanashiensis* strain A2D, *Streptomyces padanus* PMS-702, and *Streptomyces griseocarneus*, glucose proved to be the most optimal carbon source for the synthesis of antifungal polyenes (Zheng et al., 2000; Wu et al., 2008; Singh et al., 2009). Furthermore, microorganisms need the right carbon-to-nitrogen ratio for their growth and metabolism (Ray et al., 2007). A low carbon-to-nitrogen ratio can result in excessive growth, premature senescence, and autolysis of the bacteria, whereas low fermentation density is caused by a high carbon-to-nitrogen ratio that is unfavorable to the bacteria’s ability to reproduce (Pan et al., 2023). In the present study, the nitrogen source (beef extract) exhibited the highest nematicidal potential of 84%, indicating that it is a suitable nitrogen source for nematode control. Several trace elements are necessary for microbial development due to their roles as enzyme activators (Gonzalez and Stres, 2019). The most crucial trace elements for secondary metabolism are zinc, iron, and manganese (Locatelli et al., 2016). Similarly, inorganic salts provide vital mineral components for microbial development and have inhibitory effects on a variety of diseases (Miller, 2017). In the current investigation, the addition of potassium chloride and disodium hydrogen phosphate resulted in substantial increases in the nematicidal activity of fermentation broth, which was 82.54% and 81.53%, respectively.

Secondary metabolite synthesis by microbes is extremely reliant on species and strain existent, along with nutritional and cultural circumstances (Ruiz et al., 2010; Horak et al., 2019). The fermentation of microbes is a complicated, unpredictable, and uncontrolled procedure (Lombardi et al., 2020). Slight modifications to the combination of the fermentation medium and conditions may alter compound yields and the metabolic characteristics of a strain (Parekh et al., 2000; Lee et al., 2005). It is challenging to identify the optimal fermentation circumstance (Kwon et al., 2023). RSM may optimize the composition of the fermentation medium and growth conditions to promote secondary metabolite synthesis, thereby facilitating the identification of new natural active substances (Yun et al., 2018). RSM was employed in the current research to optimize the fermentation circumstances (Kuo et al., 2021). In the beginning, the Plackett–Burman method was utilized to determine major determinants impacting metabolite synthesis (Huang et al., 2018). This broad variation in nematicidal activity demonstrated the significance of medium constituent optimization (Lee and Kim, 2015). In the current investigation, glucose, peanut cake flour, and potassium chloride demonstrated significantly higher nematicidal potential and were identified as important factors for further optimization studies. Furthermore, using the Box–Behnken design, the corrected mortality of J2 ranged from 52.24% to 91.15%. Our results also agree with those obtained by Zhang et al. (2012) that RSM is a useful method for optimizing fermentation parameters because of its rapid testing cycle, excellent precision, and low testing frequency. Previous studies also revealed that optimization of fermentation increased the nematicidal potential of biocontrol agents (Abdel-Salam et al., 2018; Nguyen et al., 2021). Similarly, our study findings serve as a helpful benchmark for developing future techniques for bionematicide fermentation that are more effective and practical. Despite the positive results of this study, more investigation is required to determine the active ingredient and the efficacy of Sneb518 fermentation against *M. incognita* J2s.

## 5 Conclusion and future perspectives

The present investigation revolves around the development, evaluation, and optimization of fermentation characteristics using useful bacteria *C. beijerinckii* Sneb518 for increased nematicidal activity and biocontrol effectiveness. To the best of our knowledge, our investigation into the use of the Plackett–Burman model and RSM to assess and enhance fermentation features for the biocontrol efficacy of *C. beijerinckii* against *M. incognita* in surface and submerged fermentation processes is the first of its kind. Based on current findings, statistical analysis utilizing RSM and the Plackett–Burman design is an effective technique for elucidating and improving the interrelated impacts of the factors affecting microbial fermentation. The generated model using the Plackett–Burman design and RSM analysis is more precise and reliable than the model obtained prior to optimization in terms of exploiting the nematicidal ability of Sneb518 culture filtrates. This model will make it easier to develop certain new marketable biocontrol agents in the future by facilitating a more effective and efficient bio-nematicide fermentation procedure.

## Data Availability

The raw data supporting the conclusion of this article will be made available by the authors, without undue reservation.
